# Two-stage automated diagnosis framework for urogenital schistosomiasis in microscopy images from low-resource settings

**DOI:** 10.1117/1.JMI.10.4.044005

**Published:** 2023-08-07

**Authors:** Prosper Oyibo, Brice Meulah, Michel Bengtson, Lisette van Lieshout, Wellington Oyibo, Jan-Carel Diehl, Gleb Vdovine, Tope Agbana

**Affiliations:** a Delft University of Technology, Delft Center for Systems and Control, Faculty of Mechanical, Maritime, and Materials Engineering, Delft, The Netherlands; b University of Lagos, College of Medicine, Centre for Malaria Diagnosis, NTD Research, Training, and Policy/ANDI Centre of Excellence for Malaria Diagnosis, Lagos, Nigeria; c Leiden University Medical Centre, Department of Parasitology, Leiden, The Netherlands; d Centre de Recherches Medicales des Lambaréné, CERMEL, Lambarene, Gabon; e Delft University of Technology, Department of Sustainable Design Engineering, Faculty of Industrial Design Engineering, Delft, The Netherlands

**Keywords:** schistosomiasis diagnosis, deep learning, semantic segmentation, ellipse fitting, edge artificial intelligence

## Abstract

**Purpose:**

Automated diagnosis of urogenital schistosomiasis using digital microscopy images of urine slides is an essential step toward the elimination of schistosomiasis as a disease of public health concern in Sub-Saharan African countries. We create a robust image dataset of urine samples obtained from field settings and develop a two-stage diagnosis framework for urogenital schistosomiasis.

**Approach:**

Urine samples obtained from field settings were captured using the Schistoscope device, and *S. haematobium* eggs present in the images were manually annotated by experts to create the SH dataset. Next, we develop a two-stage diagnosis framework, which consists of semantic segmentation of *S. haematobium* eggs using the DeepLabv3-MobileNetV3 deep convolutional neural network and a refined segmentation step using ellipse fitting approach to approximate the eggs with an automatically determined number of ellipses. We defined two linear inequality constraints as a function of the overlap coefficient and area of a fitted ellipses. False positive diagnosis resulting from over-segmentation was further minimized using these constraints. We evaluated the performance of our framework on 7605 images from 65 independent urine samples collected from field settings in Nigeria, by deploying our algorithm on an Edge AI system consisting of Raspberry Pi + Coral USB accelerator.

**Result:**

The SH dataset contains 12,051 images from 103 independent urine samples and the developed urogenital schistosomiasis diagnosis framework achieved clinical sensitivity, specificity, and precision of 93.8%, 93.9%, and 93.8%, respectively, using results from an experienced microscopist as reference.

**Conclusion:**

Our detection framework is a promising tool for the diagnosis of urogenital schistosomiasis as our results meet the World Health Organization target product profile requirements for monitoring and evaluation of schistosomiasis control programs.

## 1 Introduction

Schistosomiasis is endemic in 76 countries worldwide with ∼252  million people infected and an estimated 779 million people at risk of infection.^1^ Schistosomiasis is caused by blood flukes of the genus *Schistosoma* (*S*); both *S. mansoni* (intestinal schistosomiasis) and *S. haematobium* (urogenital schistosomiasis) are endemic in Africa.^2^ Schistosomiasis presents a substantial public health and economic burden as it is a disease of poverty. In the drive to attain the World Health Organization (WHO) control and elimination targets, diagnosis for adequate monitoring of interventions and surveillance is critical.^2^^,^^3^ Recently, the WHO published the diagnostic target product profiles (TPP) for monitoring, evaluation, and surveillance of schistosomiasis control programs,^4^ which identifies development of diagnostic tests for *S. haematobium* detection as a high-risk requirement due to lack of its availability. The TPP suggests a semi-quantitative analysis, capable of providing some degree of information regarding intensity of infection, as ideal for a diagnostic test for schistosomiasis to support monitoring and evaluation.^4^ Currently, microscopy is the WHO reference standard for the diagnosis of schistosomiasis in resource-limited settings. For the detection of *S. haematobium* infection, urine samples, after filtration, sedimentation, or centrifugation, are microscopically examined for the presence of eggs.^3^ This method is operator dependent, costly, laborious, and time-consuming. Furthermore, it requires expertise, which means microscopy skills need to be gained and maintained, which can be an economic challenge, particularly in remote rural communities.^3^ There is also the risk of visual health complications among microscopists resulting from excessive workload due to the low ratio of trained microscopists to samples for analysis in endemic regions.^5^ Hence, a field adaptable, rapid, and easy-to-use automated diagnosis is relevant for the prompt detection of cases, which will facilitate mapping and monitoring of interventions.^4^ Recent advances in opto-mechanics and opto-electronics have rapidly transformed the field of biomedical optics. Optical imaging technologies, such as conventional light microscopes, are being redesigned to integrate and miniaturize portable light microscopes for use at the point of care.^6^^–^^9^ Although these technologies are readily available in high-income countries, unfortunately, nearly all schistosomiasis cases are seen in low-resource regions of low-income countries, significantly justifying the need for cost-effective and easy-to-use smart diagnostic technologies. In this work, we address these challenges by first increasing the size of the *S. haematobium* (SH) dataset in our previous work^10^ from 5198 to 12,051 images of clinical samples.^11^ We carry out detection and counting of *S. haematobium* eggs present in each image by proposing a two-stage framework consisting of a DeepLabv3 with MobilenetV3 backbone deep convolutional neural network^12^ trained on the SH dataset using a transfer learning approach. The second stage of our proposed framework is a refined segmentation and egg counting procedure, which adapts the region-based fitting of overlapping ellipses^13^ to efficiently separate the boundaries of overlapping eggs in the image. Finally, the detected isolated eggs are screened for the presence of an egg, which meets the defined boundary condition before the sample can be determined as positive/negative diagnosis. We further demonstrate the robustness and applicability of the proposed framework for field diagnoses of urogenital schistosomiasis by implementing our framework on an Edge AI system (Raspberry Pi + Coral USB accelerator) and testing 65 clinical urine samples obtained in a field settings in Nigeria. The main contributions of this work can be summarized as follows.

1. A large-scale *S. haematobium* egg dataset of 12,051 images captured in field settings is created with respective manually annotated mask images. The dataset contains images with artifacts, such as crystals, glass debris, air bubbles, and fibres.
2. A *S. haematobium* egg detection framework consisting of the DeepLabv3 with MobileNetV3 backbone deep convolutional neural network, trained using transfer learning approach for semantic segmentation of the eggs. This effectively segments transparent eggs in noisy images taken in the field. The framework also separates overlapping eggs using a refined segmentation algorithm resulting in a more accurate egg count.
3. The implementation and testing of the *S. haematobium* egg on an Edge AI system to demonstrate its field applicability for the diagnosis of schistosomiasis in low-resource settings.

## 2 Related Work

A pioneering study on the identification and classification of human helminth eggs based on computer vision algorithms was carried out by Ref. 14. However, their focus was on helminth eggs found in microscopic faecal samples. Subsequent works^15^^–^^18^ included the detection *S. haematobium* eggs found in urine but only in images pre-captured by professional clinical operators mostly with isolated and non-overlapping eggs in the field of view (FoV) images. Regarding the detection of *S. haematobium* eggs in microscopy images of urine from field settings, these images contain many artifacts with morphological and textural similarity to eggs, such as crystals, glass debris, air bubbles, fabric fibres, and human hair. This makes it difficult to achieve high accuracy using traditional AI methods, which detect objects in the images based on some threshold value or discontinuous local features of an image. The *S. haematobium* eggs are oval-shaped structures (110 to 170  μm long and 40 to 70  μm wide) with a thick transparent capsule and a sword-shaped protrusion known as the terminal spine located at the narrow end of the egg. Detecting an egg is challenging due to its similar appearance to its surroundings. Automated detection of an isolated *S. haematobium* egg by thresholding the cross-correlation coefficient of two sets of invariant moments for both a reference and sample image was performed by Ref. 19. However, this method had poor performance in noisy images and hence cannot be used for *S. haematobium* eggs detection in field settings.

Recently, deep learning algorithms were used by Ref. 20 to solve the challenges of *S. haematobium* egg detection in images captured in field settings. Using transfer learning, they compared RetinaNet,^21^ MobileNet,^22^ and EfficientDet^23^ architectures pre-trained on the COCO 2017 dataset.^24^ They retained the feature extraction layers and fine-tuned the dense layers of these models to detect *S. haematobium* eggs as a single class. The RetinaNet architecture had improved egg detection performance with egg counts closely related to manual egg counts obtained by a trained user. It was also able to detect isolated eggs and reject other debris from a crowded FoV. However, air bubbles were incorrectly classified as eggs, and the automated detection of eggs aggregated in large clumps with other eggs or debris remained a challenge. In our previous work, we developed a low-cost automated digital microscope (Schistoscope V5.0) with AI for the detection *S. haematobium* eggs,^10^ and we reported the results from a field validation study in Nigeria.^11^ A U-Net model^25^ trained with the *S. haematobium* dataset consisting of 5198 images captured from both clinical and spiked urine samples was used for automated egg detection. Although we achieved a high diagnostic sensitivity of 87.3%, the diagnostic specificity was low (48.9%). This was due to the high number of false positives by the U-Net architecture and the inability of the segmented pixel area-based linear model to filter out incorrectly segmented eggs while counting.

All these studies show that deep learning is a promising approach for the automated diagnosis of urogenital schistosomiasis. However, developing a model that is field applicable requires a robust dataset of images with varying degrees of urine artifacts from field settings. Also the separation of overlapping eggs for improved estimation of infection intensity has remained a challenge. This paper proposes a two-stage framework to solve these challenges.

## 3 Methods

**Fig. 1:**
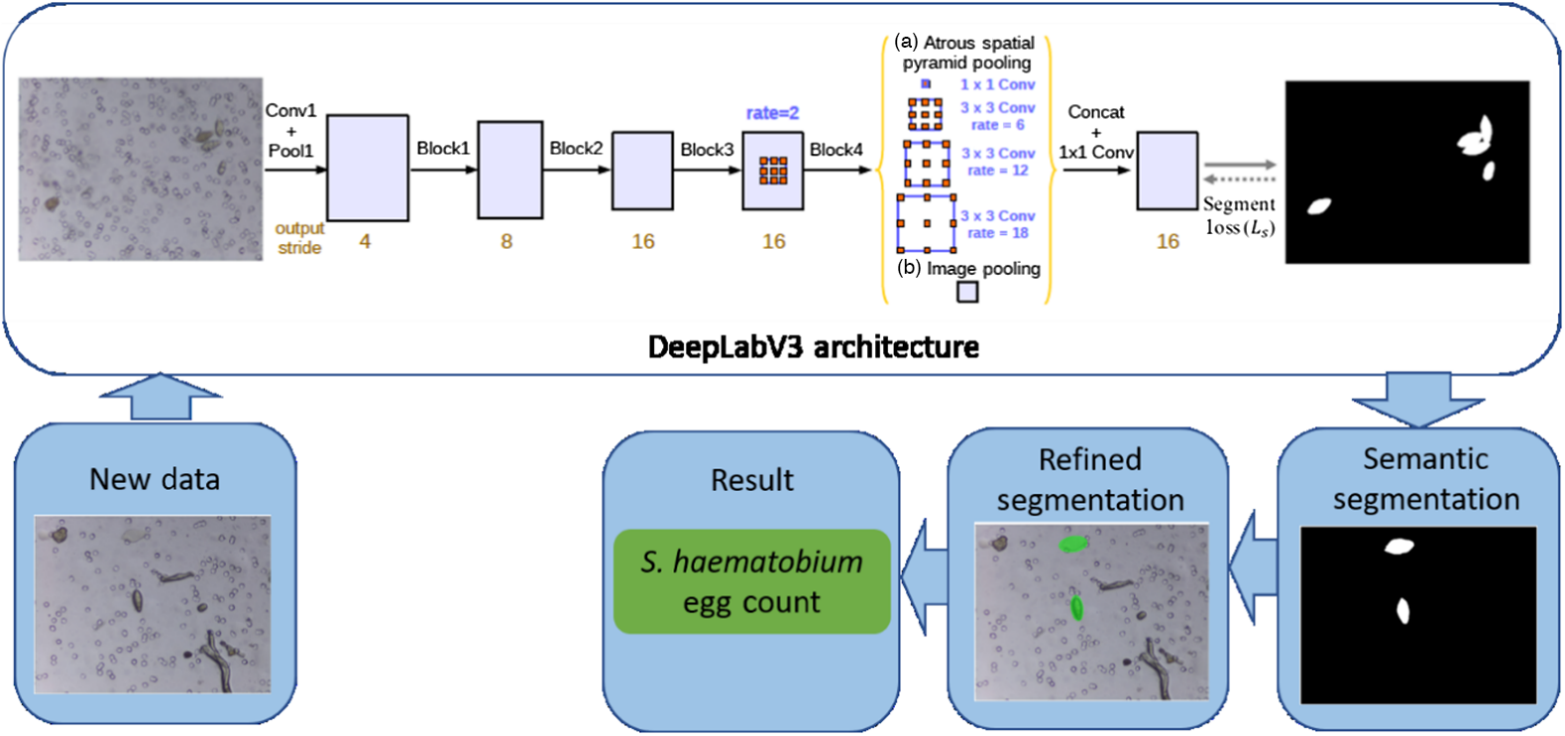
Schematics of the proposed two-stage diagnosis framework urogenital schistosomiasis with DeepLabV3-MobileNetV3 deep learning architecture for semantic segmentation of eggs and refined segmentation for overlapping eggs separation and count.

To meet the WHO TPP requirements for a diagnostic test for schistosomiasis, the proposed urogenital schistosomiasis diagnostic framework consists of two stages (Fig. 1). The first stage involves the semantic segmentation of candidate *S. haematobium* eggs in captured images. The segmentation results are further refined in the second stage by ellipse fitting and morphological filtering of the segmented regions. The two-stage framework minimizes false positive detection that enables a high diagnostic specificity, which is a requirement for diagnostic tools for monitoring and evaluation of schistosomiasis control programs and determining transmission interruption.

### 3.1 Sample Image Capture and S. haematobium Egg Annotation

The details of the Schistoscope’s mechanical precision and optical quality are described in our previous work.^10^ The Schistoscope optical system consists of a 4× magnification microscope objective and a Raspberry Pi High-Quality Camera Module V2.1 equipped with a Sony IMX477R camera sensor. The camera sensor has a pixel-pitch of 1.55  μm and registers an image size of 2028×1520  pixels. The device consists of an autofocusing and an automated slide scanning system. For urine filtration, we made use of a 13 mm filter membrane, which results in 117 image grid segments per sample when scanned by the device. The *S. haematobium* eggs in the captured images used for training and development testing were manually annotated by an expert parasitologist using the coco annotator tool.^26^ The annotation process is highlighted as follows.

1. Annotation of the exact boundary pixels of the *S. haematobium* eggs was not strictly enforced due to the limitation posed by the size of the eggs.
2. The pixel values of the filter membrane and artifacts in the ground-truth image were labeled as “0” (background) and the eggs as “1” (foreground).
3. There were few *S. mansoni* eggs found in the images of the clinical urine samples and their pixel values were labeled as “1” (foreground).
4. Pixels of partially cut eggs at the edges of the images were labeled as “1” (foreground).
5. The region of the eggs covered by artifacts was labeled as “0” (background).

### 3.2 Stage 1: Semantic Segmentation of S. haematobium Eggs

#### 3.2.1 Transfer learning using DeepLabv3-MobileNetV3

In transfer learning, a model trained on one task is repurposed to another related task, usually by some adaptation toward the new task. This approach is mainly useful for tasks where enough training samples are not available to train a model from scratch, such as medical image classification for neglected tropical diseases or emerging diseases.^27^ To overcome the limited data sizes, transfer learning was used to retrain the DeepLabv3-MobileNetV3^12^ model for semantic segmentation of candidate *S. haematobium* eggs using the SH dataset. DeepLabv3 is a semantic segmentation architecture that was developed to handle the problem of segmenting objects at multiple scales. Modules are designed, which employ atrous convolution in cascade or in parallel to capture multi-scale context by adopting multiple atrous rates. We initialize the model with weights obtained from the pre-trained model on a subset of COCO train2017, on the 20 categories that are present in the Pascal VOC dataset.^28^ Since our case consists of two output classes (background and foreground), we replace the 21-output channel convolutional layer with a single output-channel convolutional layer. The weights of all layers of the model are then updated during the training stage.

#### 3.2.2 Loss function

The model was trained using the Dice similarity coefficient (DSC) loss function,^29^ which is widely used in medical image segmentation tasks to address the data imbalance problem between foreground and background:

$$L_{\mathrm{DSC}}=\frac{2\sum_{x,y}(S_{i,x,y}\times G_{i,x,y})}{\sum_{x,y}S_{i,x,y}^{2}+\sum_{x,y}G_{i,x,y}^{2}},$$ (1)

where $S_{i,x,y}$ and $G_{i,x,y}$ refer to the value of pixel (x,y) in the segmentation result $S_{i}$ and ground truth $G_{i}$, respectively.

### 3.3 Stage 2: Refined Segmentation of S. haematobium Eggs

To solve the challenge of obtaining accurate egg counts in the occurrence of false positives or overlapping and clustered eggs, we adopted a refined segmentation procedure, which involves fitting ellipses over the region of interest in the binary output image of the semantic segmentation. The refined segmentation algorithm as shown in Algorithm 1 operates in a number of steps, which can be summarized as follows.

**Algorithm 1:** The refined segmentation algorithm

1 **Input**: Binary segmentation mask image I
2 **Output**: Set of ellipse $E_{I}^{*}$, egg count $N_{I}^{*}$
3 $N_{I}^{*}=0$
4 $E_{I}^{*}=Ø$
5 **for each** region image R∈I **do**
6 $N_{R}^{*}=0$
7 $E_{R}^{*}=Ø$
8 $AIC_{R}^{*}=\infty$
9 $N_{lb},N_{ub}=ComputeBoundary(A_{R})$
10 $N_{R}=N_{lb}$
11 **repeat**
12 $E_{R}=FitEllipse(R,N_{R})$
13 $AIC_{R}=ComputeAIC(R,U_{E})$
14 **if** $AIC_{R}<AIC_{R}^{*}$ **then**
15 $N_{R}^{*}=N_{R}$
16 $AIC_{R}^{*}=AIC_{R}$
17 $E_{R}^{*}=E_{R}$
18 $N_{R}=N_{R}+1$
19 **until** $N_{R}=N_{ub}$
20 $E_{I}^{*}=union(E_{I}^{*},E_{R}^{*})$
21 $N_{I}^{*}=N_{I}^{*}+N_{R}^{*}$
22 **end**
23 **return** $N_{I}^{*},E_{I}^{*}$
**Legend**
I: Binary segmentation mask image
$E_{I}^{*}$: Optimal set of ellipses for I
$N_{I}^{*}$: Optimal number of ellipses for I
R: Segmented egg region image
$N_{R}^{*}$: Optimal number of ellipses for R
$E_{R}^{*}$: Optimal set of ellipses for R
$AIC_{R}^{*}$: Optimal Akaike Information Criterion for R
$N_{lb}$: Lower boundary for N
$N_{ub}$: Upper boundary for N

#### 3.3.1 Optimization problem formulation

We assume a binary image I that represents the segmentation mask output of the DeepLabV3-MobileNetV3 deep neural network model. The binary image may contain one or more sliced binary region image R, which has the same size as the bounding box. This region image R represents a segmented isolated or overlapping eggs. A pixel p of R belongs to either the foreground FG (R(p)=1) or the background BG (R(p)=0). The area $A_{R}$ of the segmented egg is given by

$$A_{R}=\underset{p\in \mathrm{FG}}{\sum }R(p).$$ (2)

We also assume a set $E_{R}$ of $N_{R}$ ellipses are fitted over the region image R. The binary image $U_{E}$ is defined such that $U_{E}(p)=1$ at point p that is inside any of the ellipse $E_{R,i}$; otherwise $U_{E}(p)=0$. Also we define the coverage $\alpha (E_{r})$ of the segmented eggs by the given set of ellipses $E_{R}$ as

$$\alpha (E_{r})=\frac{1}{A_{R}}\underset{p\in \mathrm{FG}}{\sum }R(p)U_{E}(p).$$ (3)

Essentially, $\alpha (E_{r})$ is the percentage of the segmented eggs that are under some of the ellipse in $E_{R}$. Let the sum of the areas of all the ellipses be denoted by $\left|E_{R}|=\sum (i=1)|E_{R,i}\right|$ and let $C(E_{R})$ denote the coverage area by all the ellipses:

$$C(E_{R})=\sum_{p\in R}U_{E}(p).$$ (4)

It should be stressed that $C(E_{R})<|E_{R}|$, with the equality holding in the case that all ellipses are pairwise disjoint. This is because in case of two overlapping ellipses, $\left|E_{R}\right|$ counts the area of their intersection two times, while $C(E_{R})$ does not. Similar to the work of Ref. 13, we want to maximize the shape coverage $\alpha (E_{R}^{*})$ with a set of ellipses $E_{R}^{*}$ whose covered area by all ellipses $C(E_{R}^{*})$ is as close as possible to $A_{R}$:

$$E_{R}^{*}=\arg\;\underset{E_{R}}{\max}\;\alpha (E_{r})\;\mathrm{s.t.}\;C(E_{R})=A_{R}.$$ (5)

We defined a model complexity measure the ratio of the area $A_{R}$ of the segmented region to experimentally observed average area of segmented isolated egg $A_{R}^{*}$:

$$C=\frac{A_{R}}{A_{R}^{*}}.$$ (6)

To estimate the optimal number $N_{R}^{*}$ of ellipses in a segmented egg region image R, a trade-off between the egg coverage $\alpha (E_{r})$ and the model complexity C is optimized by employing the Akaike information criterion (AIC).^30^ The AIC-based model selection criterion amounts to the minimization of the quantity:^13^

$${\mathrm{AIC}}_{R}=C\;\ln(1-\alpha (E_{r}))+2N_{R}.$$ (7)

This minimizes the error in egg count as intuitively the complexity is proportional to the area of the segmented eggs.

#### 3.3.2 Extracting segmented egg regions

First, connected components in the binary segmentation mask image are extracted and binary region image R, which has the same size as bounding box of the connected component is created. If area $A_{R}$ of the region image (i.e., the number of pixels in the segmented egg region) is less than the defined area threshold $A_{t}h$, then the detected region is classified as noise; otherwise, we solve for the optimal number of ellipses as described in the next section.

#### 3.3.3 Initializing ellipses solutions

For defined number $N_{R}$ of ellipses in a segmented egg region image R, we initialize the ellipse hypotheses using k-means clustering this defines a set $E_{R}$ of clusters, which are circular in shape with hard cut-off borders where each pixel is strictly allocated to one cluster. The cluster centers are the mean vector of the points belonging to the respective cluster, while the diameters are the maximum Euclidian distances of the cluster members from their respective cluster centers.

#### 3.3.4 Optimizing ellipses solutions

To obtain a more complex, ellipsoid shapes with soft cut-off borders (i.e., overlapping ellipses) which closely describes the shape of *S. haematobium* eggs, the ellipse hypotheses is evolved using the Gaussian mixture model expectation maximization (GMM-EM) algorithm to finetune the parameters of the initialized set $E_{R}$ of clusters with the best coverage $\alpha (E_{r})$ of the given segmented egg region. This is achieved by expectation-step and the maximization-step iteratively of the GMM-EM algorithm. The log likelihood function is maximized until the GMM-EM algorithm converges. A detailed explanation of the GMM-EM algorithm can be found in the work of Refs. 31 and 32.

#### 3.3.5 Solving for the optimal number of ellipses

Different models (i.e., solutions involving different numbers $N_{R}$ of ellipses) for a segmented egg image region are evaluated based on the AIC criterion [defined in Eq. (7)] that balances the trade-off between model complexity and approximation error. To minimize ${\mathrm{AIC}}_{R}$, the refined segmentation algorithm increments the number of candidate ellipses $N_{R}$ starting from a lower boundary, $N_{l}b=0.6\text{ceil}(C)$, with a step size of 1. At each value of $N_{R}$, the set $E_{R}$ of clusters is first initialized by k-means clustering (described in Sec. 3.3.3) and then evolved using the GMM-EM algorithm (described in Sec. 3.3.4). This process continues until $N_{R}$ is equal to the upper boundary, $N_{u}b=1.1\text{ceil}(C)$. In each iteration, the ${\mathrm{AIC}}_{R}$ criterion is computed. The lower and upper boundaries of the number of ellipses are formulated using the complexity measure C, derived from prior knowledge about the average pixel area of the *S. haematobium* eggs. This helps to reduce the search space for the optimal number of ellipses. From all possible models (involving from $N_{l}b$ to $N_{u}b$), the refined segmentation algorithm reports as the optimal solution as the set of ellipses $E_{R}^{*}$ with the minimum ${\mathrm{AIC}}_{R}$.

#### 3.3.6 Morphological filtering of detected isolated eggs

To reduce these false positives diagnosis caused by pixels of artifacts, such as crystals wrongly segmented as isolated egg, we introduced two linear inequality constraints, which are functions of the area of the detected ellipse $\left|E_{R}\right|$ and overlap coefficient $\mathrm{OC}(R,U_{E})$ defined by the following ratio:

$$\mathrm{OC}(R,U_{E})=\frac{R\cap U_{E}}{\min(R,U_{E})}.$$ (8)

These inequality constraints are derived experimentally and only applied to segmented regions with a single fitted ellipse for determination of diagnosis result. This improves the specificity of the algorithm by accepting only regions that fall within an experimentally defined boundary region as candidate *S. haematobium* eggs while discarding the others as a false positive prediction.

## 4 Dataset and Implementation Details

### 4.1 Dataset Description

**Fig. 2:**
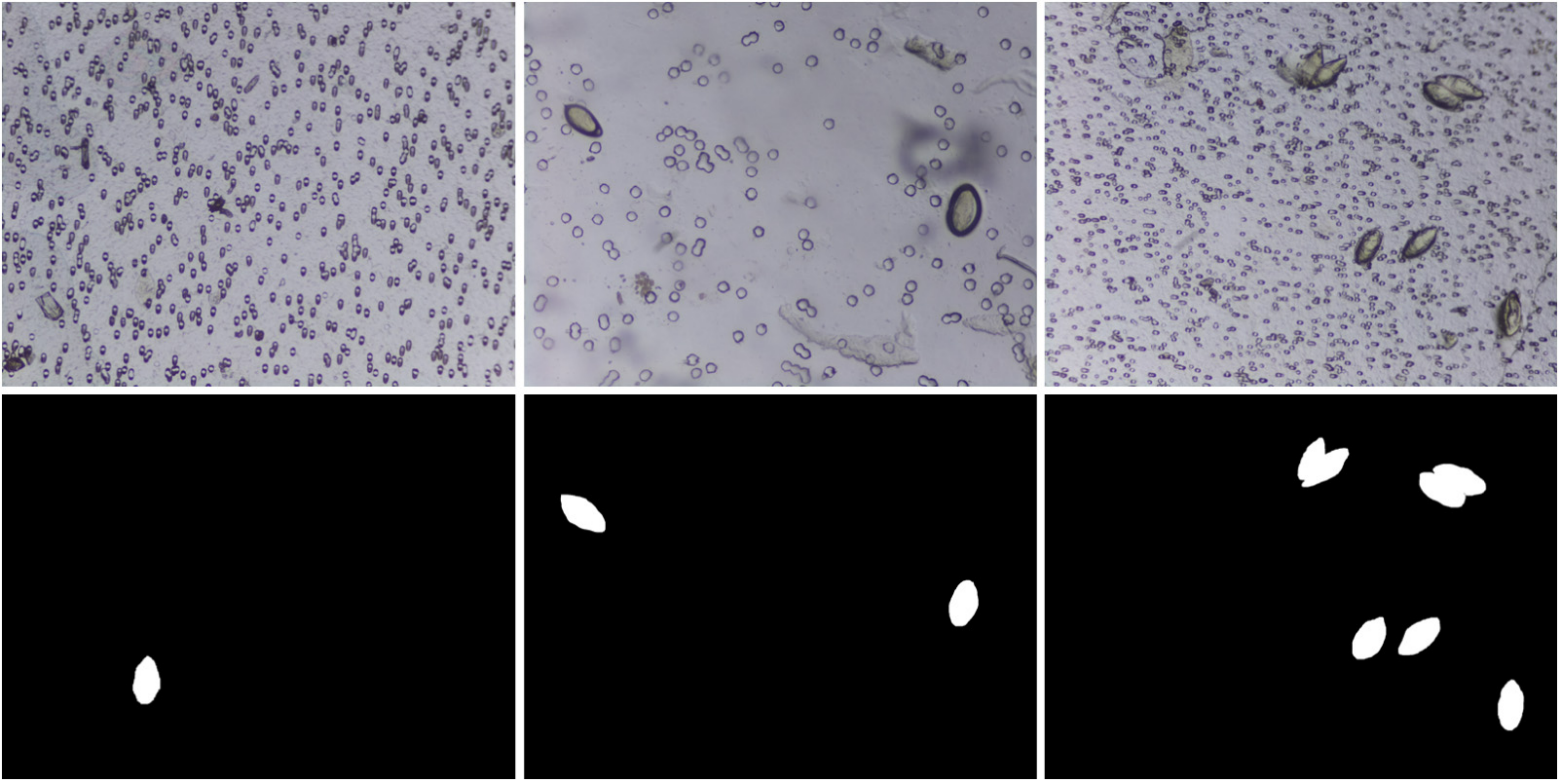
Example of sample images that are easy-to-identify eggs having glass slides and filter membranes as background and their respective ground truth images.

**Fig. 3:**
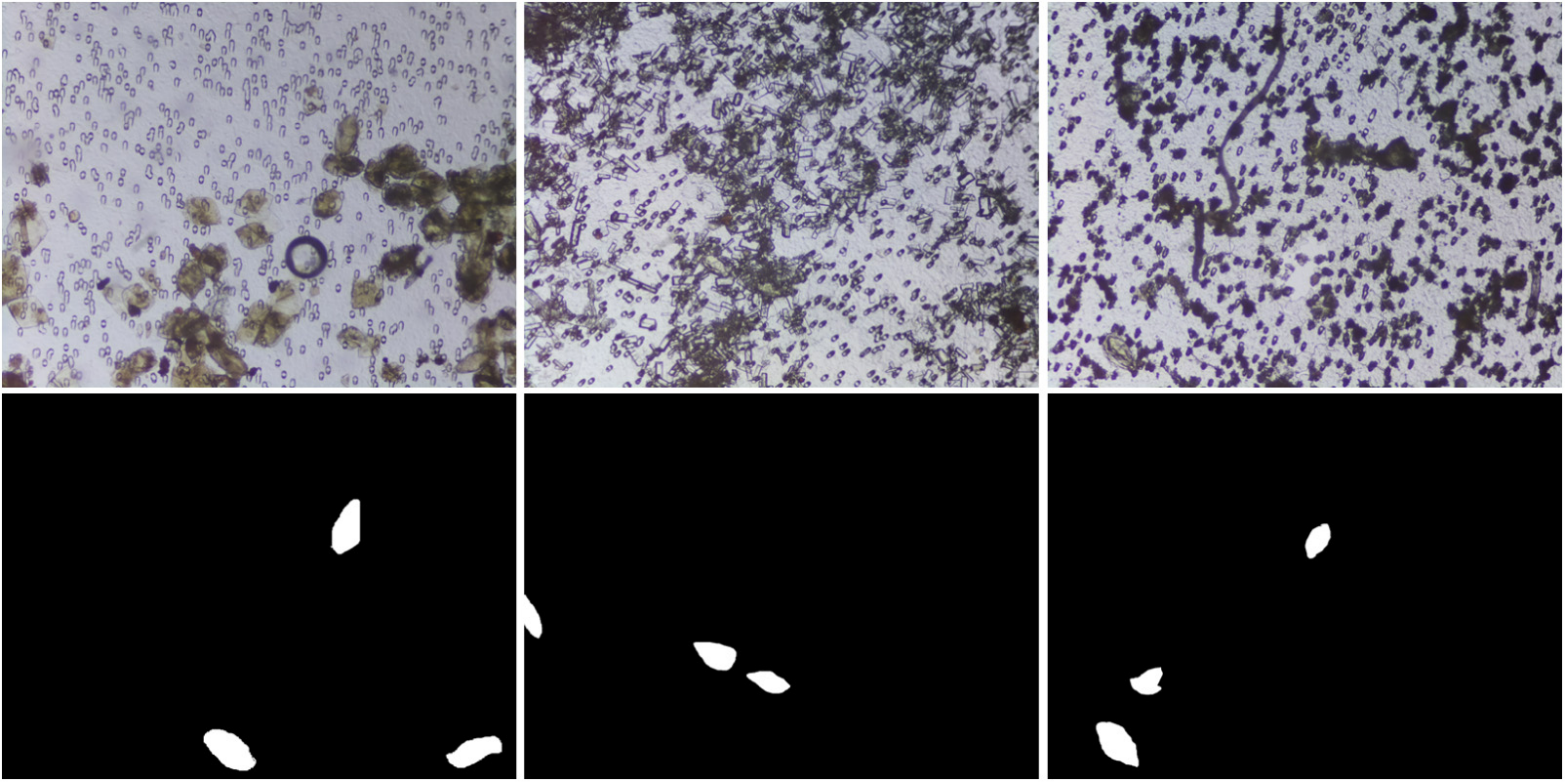
Example of sample images that are difficult-to-identify eggs with artifacts, such as crystals, glass debris, air bubbles, and fabric fibers in the background, and their respective ground truth images.

A total of 103 captured urine samples were used for the creation of the SH dataset. The SH dataset was used for training and development testing of the DeepLabV3-MobileNetV3 deep neural network model, while a separate set of 65 captured urine samples referred to as diagnosis test dataset was used for testing the developed framework for urogenital schistosomiasis diagnosis. The images were captured from urine samples collected in a rural area in central Nigeria with the Schistoscope V5.0.^11^ The size of the captured images is 1520×2028  pixels. The details of the sample collection and preparation process are described in our previous works.^10^^,^^11^ The procedure followed in capturing and annotation of the *S. haematobium* eggs in images is described in Sec. 3. A summary of the SH image dataset is shown in Table 1. It consists of 12,051 images of clinical urine samples and their respective mask images. There are 17,799 annotated *S. haematobium* eggs in 2997 captured FoV images. The dataset consists of images that are easy to identify eggs (Fig. 2) without the presence of artifacts in the background, as well as images that are difficult to analyse (Fig. 3) with backgrounds containing artifacts, such as crystals, glass debris, air bubbles, fabric fibres, and human hair, which makes egg identification difficult. The SH dataset is split into 80% (9641 images) and 20% (2410 images) for training and development testing, respectively. To our best knowledge, this SH dataset is the largest robust dataset focused on *S. haematobium* egg images captured in a field setting.

**Table 1:** Number of images per category in the SH dataset.

Split	Positive images	Negative images	Total
Training set (80%)	2420	7221	9641
Test set (20%)	577	1833	2410
Total	2997	9054	12,051

### 4.2 Implementation Details

The training of the DeepLabv3-MobileNetV3 model was performed using the Pytorch framework^33^ on NVIDIA A100-SXM4-40GB GPU. All images were pre-processed by centring and normalizing the pixel density per channel. We fine-tuned the model for 100 epochs. The batch size is set to 8, and ADAM optimizer is used to optimize the Dice loss function, with an initial learning rate of $1\times 10^{-4}$. We employ a “poly” learning rate policy,^12^ where the initial learning rate is multiplied by ${\left(1-\mathrm{iter}/(\max_{i}\mathrm{ter})\right)}^{\text{power}}$ with power=0.9. All images were down sampled to 507×676 before being fed to the neural network.

To demonstrate the field applicability of the two-stage framework in low-resource settings, we performed the development testing and diagnosis testing on a Raspberry Pi 4 model B using a Coral USB accelerator. To perform semantic segmentation on the Edge AI system, we converted the DeepLabv3-MobileNetV3 model from Pytorch to TensorFlow lite.^34^ This was done by first exporting the Pytorch model in Open Neural Network Exchange (ONNX) format. The ONNX model is then converted to TensorFlow before the final conversion from TensorFlow to TensorFlow Lite. The refined segmentation algorithm was implemented on the Raspberry Pi.

### 4.3 Evaluation Metrics

We evaluated the performance of semantic segmentation of *S. haematobium* egg by comparing the DeepLabV3-MobileNetV3 segmentation, which are the prediction results with a ground truth (GT) that was manually annotated by a trained parasitologist using the pixel accuracy (PA):

$$\mathrm{PA}=\frac{1}{n}\overset{n}{\underset{i=0}{\sum }}1_{\left({\mathrm{GT}}_{i}={\mathrm{DS}}_{i}\right)}.$$ (9)

We also compared the semantic segmentation performance using DSC and Jaccard similarity coefficient (JAC), which are widely used in evaluating medical segmentation algorithms:

$$\mathrm{DSC}=2\frac{\left|\mathrm{GT}\cap \mathrm{DS}\right|}{\left|\mathrm{GT}|+|\mathrm{DS}\right|},$$ (10)

$$\mathrm{JAC}=\frac{\left|\mathrm{GT}\cap \mathrm{DS}\right|}{\left|\mathrm{GT}\cup \mathrm{DS}\right|}.$$ (11)

Although the diagnostic performance of our two-stage diagnosis framework was evaluated by employing three metrics, precision, sensitivity, and specificity, which are commonly used for evaluating diagnostic devices:

$$\text{precision}=\frac{\mathrm{TP}}{\mathrm{TP}+\mathrm{FP}},$$ (12)

$$\text{sensitivity}=\frac{\mathrm{TP}}{\left(\mathrm{TP}+\mathrm{FN}\right)},$$ (13)

$$\text{specificity}=\frac{\mathrm{TN}}{\left(\mathrm{TN}+\mathrm{FP}\right)},$$ (14)

where TP, FP, TN, and FN are true positive, false positive, true negative, and false negative samples, respectively.

## 5 Experiments and Results

### 5.1 DeepLabV3-MobileNetV3 S. haematobium Egg Semantic Segmentation

To determine the applicability of the framework on the Edge AI system in low-resource settings with no internet connectivity, we implemented and evaluated its performance on a Raspberry Pi 4B with Coral USB accelerator. We evaluate the DeepLabV3-MobileNetV3 deep learning model for the semantic segmentation of *S. haematobium* eggs using the development test dataset. As shown in Table 2, the deep learning model achieved a segmentation accuracy of 99.69%. It is, however, important to note the existence of a very high imbalance between the foreground and background pixels in the images, which could hamper the segmentation accuracy. While using the Jaccard and dice coefficient as performance metric, the model obtained 85.30% and 87.20%, respectively. However, the average inference time per image was 7.13 s with a model size of 7.13 MB. We considered the inference time too high given the need to process 117 images per sample diagnosis. To reduce the processing time on the Edge AI system, we optimized the DeepLabV3-MobileNetV3 deep learning model using post-training quantization on TensorFlow. The optimized model was applied to the development test dataset. We observed a significant reduction in inference time and model size (2× and 4×, respectively) with little effect (about 1% reduction) in the Jaccard and Dice coefficient metric. However, the segmentation accuracy remained the same. All further experiments in the work were carried out using the optimized model.

**Table 2:** Performance of DeepLabV3-MobileNetV3 for semantic segmentation of *S. haematobium* eggs.

	PA	JAC	DSC	Model size (MB)	Inference time (s)
Base model	99.69	85.30	87.20	42.1	7.13
Optimized model	99.69	84.64	86.55	11.1	4.39

**Fig. 4:**
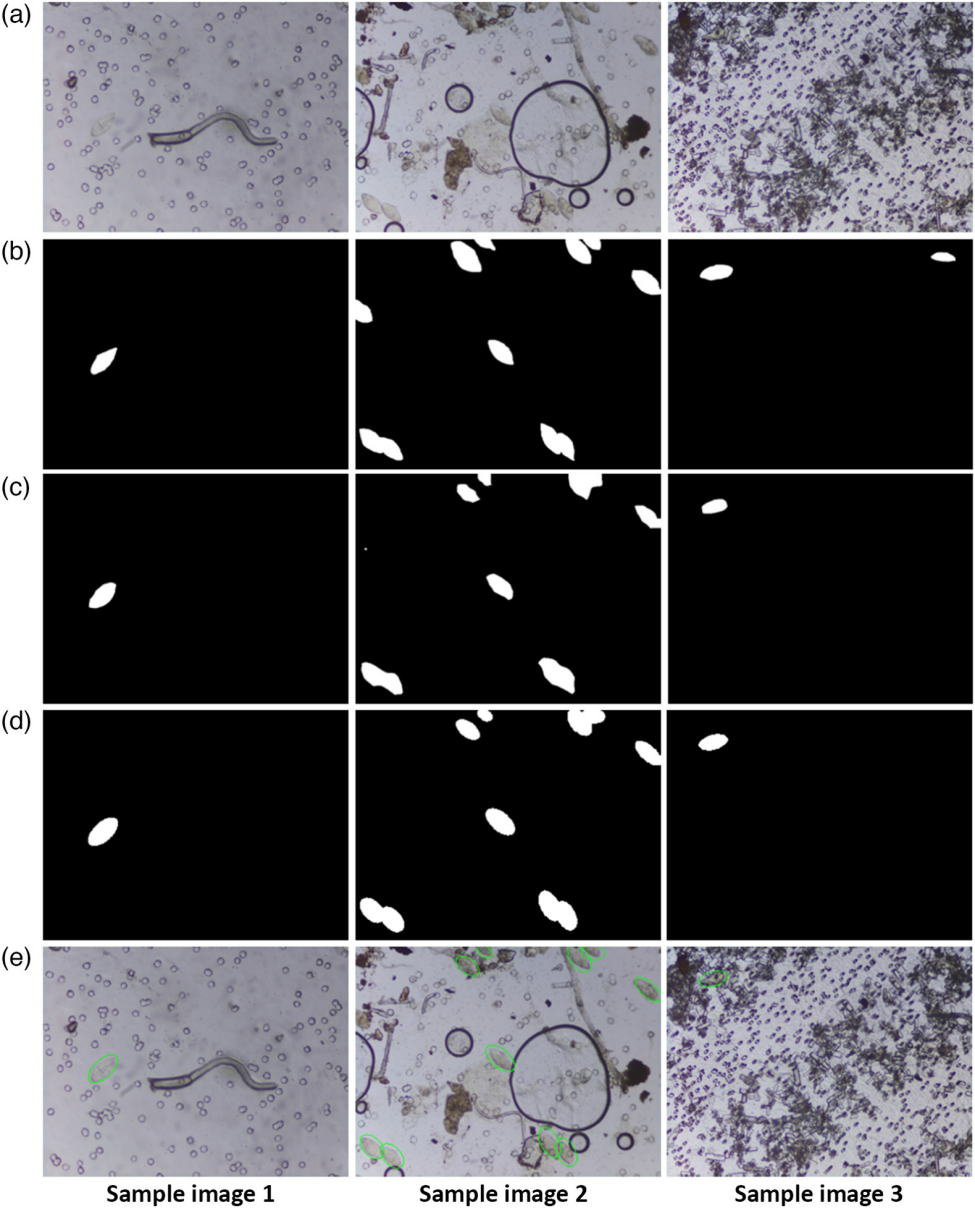
Visual performance of developed framework on sample images from the dataset. Schistoscope (a) captured and (b) ground truth images. The output mask images of (c) DeepLabV3-MobileNetV3 segmentation and (d) refined segmentation. (e) The result image with detected eggs.

The visual performance of the segmentation model is shown in Fig. 4(c). We observed that the model detected eggs in images heavily cluttered with artefacts, such as crystals and other particles (sample image 3). It also detected highly transparent *S. haematobium* eggs (sample image 1) present in the captured images. Partially cut eggs on the edge of the images and overlapping eggs were also detected as observed in sample image 2. However, the boundaries in the overlapping eggs are not clearly segmented.

### 5.2 Refined Segmentation and Egg Count

**Fig. 5:**
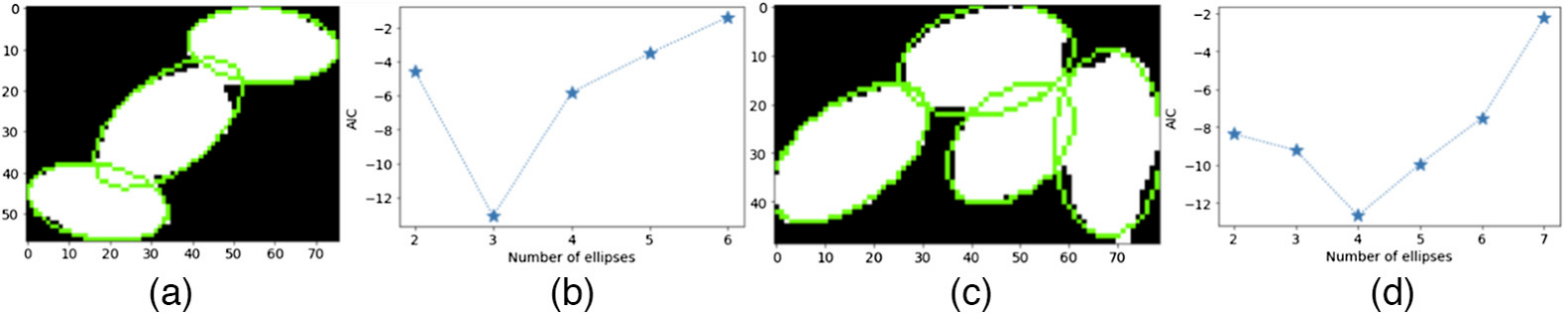
(b), (d) Estimated AIC criteria values for different number of ellipses fitted on the (a), (c) region images with optimal ellipses are highlighted in green.

In the second stage of our framework, we applied a refined segmentation algorithm on the output segmentation mask image of the DeepLabV3-MobileNetV3 deep learning model as described in Sec. 3.2. From Fig. 4(d), we observed that the refined segmentation steps fills-in eggs pixels missed in the deep learning semantic segmentation stage. This improves the visual perceptibility of the eggs in the segmentation mask image especially in regions with overlapping eggs as seen in sample image 2. Figure 5 shows example regions with overlapping eggs in the deep learning segmentation mask image. We observed that the correct number of eggs in Figs. 5(a) and 5(c) are equivalent to the optimal AIC criterion values in Figs. 5(b) and 5(d), respectively. The refined segmentation stage is able to separate overlapping eggs thus improving the accuracy of determining the infection intensity of the sample.

**Fig. 6:**
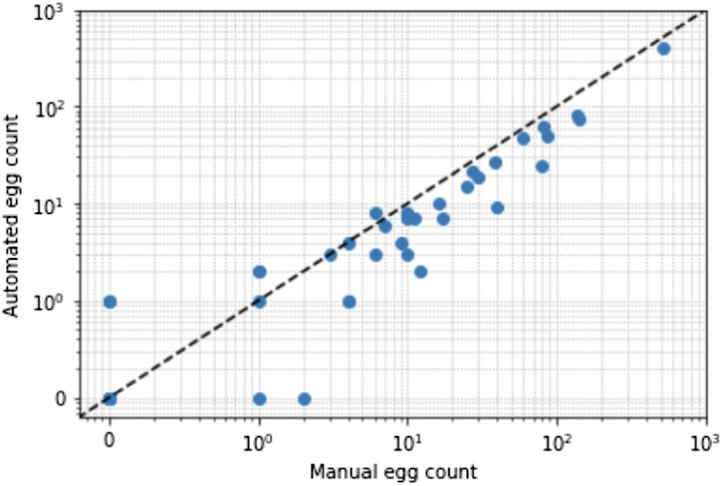
Logarithmic scale scatter plot of infection intensity per 10 mL urine sample. The manual egg count obtained by a microscopist manually counting the eggs in the diagnosis test image dataset is used as a reference, whereas the automated egg count is obtained using the developed framework.

Figure 6 shows the scatter log-scale plots of the automated egg counts versus the manual egg count (i.e., egg count by an experienced microscopist) of samples in the diagnosis test dataset. Although we observed that the predicted egg counts were mostly under the 1:1 line, this signifies underprediction especially in samples with high egg counts. However, the manual and automated egg counts are highly correlated in samples with both low and high egg counts, which indicate the applicability for the developed framework in determining infection intensity of a sample.

### 5.3 Urogenital Schistosomiasis Diagnosis

**Fig. 7:**
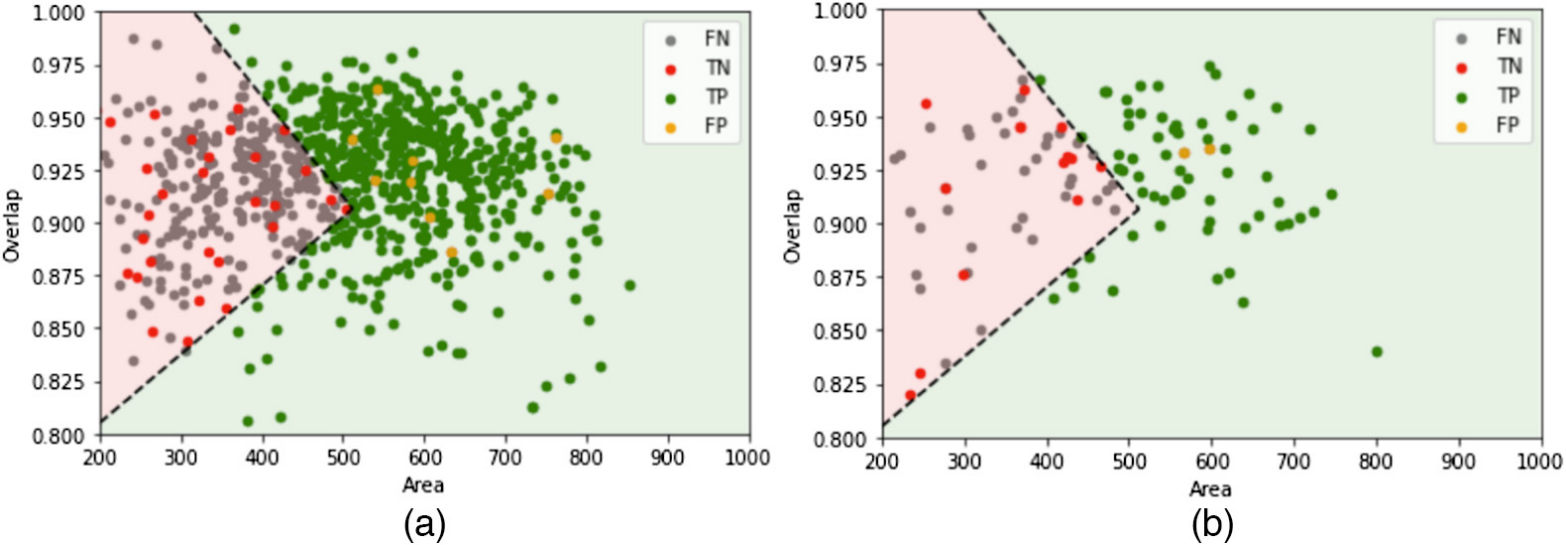
Boundary conditions to determine a sample as positive or negative diagnosis applied to images from (a) the development test dataset and (b) diagnosis test dataset. Samples are determined as positive if an egg in any of the 177 FoV images is detected in the green region.

A 10 mL urine sample consists of 117 FoV images when filtered with a 13 mm membrane and captured by the Schistoscope. For a sample to be determined as true negative diagnosis, the 117 FoV images must contain no false positives. We experimentally defined boundary conditions for the detected isolated eggs using inequality functions, defined by the overlap and area of the fitted ellipse as shown in Fig. 7. The boundary conditions are defined by $\mathrm{OC}\geq -4.75\times 10^{-4}|E_{R}|+1.15$ and $\mathrm{OC}\leq 3.25\times 10^{-4}|E_{R}|+0.74$, where OC is the overlap and $\left|E_{R}\right|$ is the area of fitted ellipse The experiment was carried out using images from the development test dataset [Fig. 7(a)], and boundaries were found to hold also in images from the diagnosis test dataset [Fig. 7(b)]. A sample was determined as positive diagnosis if an isolated egg is detected in the set of 117 FoV images, which satisfies the defined boundary conditions (an egg is detected in the green region of Fig. 7). Otherwise, the sample is determined as negative diagnosis. We observed that most of the false negatives in Fig. 7 (gray markers) were broken or partly captured eggs found at the edges of the image, whereas the false positives (yellow markers) are artefacts that are very similar in appearance to a *S. haematobium* egg.

The diagnostic performance of the developed framework is shown in Table 3. We observed a significant improvement in diagnosis specificity (from 72.73% to 93.94%) and precision (from 77.50% to 93.75%) when the boundary conditions are applied in determining the sample diagnosis. However, a reduction in the diagnosis sensitivity is observed. This is due to some samples with very low infection intensities (eggs per 10 mL of urine ≤2) not having any detected eggs, which meet the boundary constraint represented by the green region of Fig. 7(b). McNemar’s test returned a p-value of 0.008, which indicates a statistically significant difference between both methods (p-value<0.05). Also we achieved a 7.39% and 92.11% performance improvement in diagnosis sensitivity and specificity, respectively, compared to our previously published work.^11^

**Table 3:** Diagnostic performance of developed framework on the diagnosis test dataset.

	Sensitivity	Specificity	Precision
Without boundary conditions	96.88	72.73	77.50
With boundary conditions	93.75	93.94	93.75

### 5.4 Computational Time

**Fig. 8:**
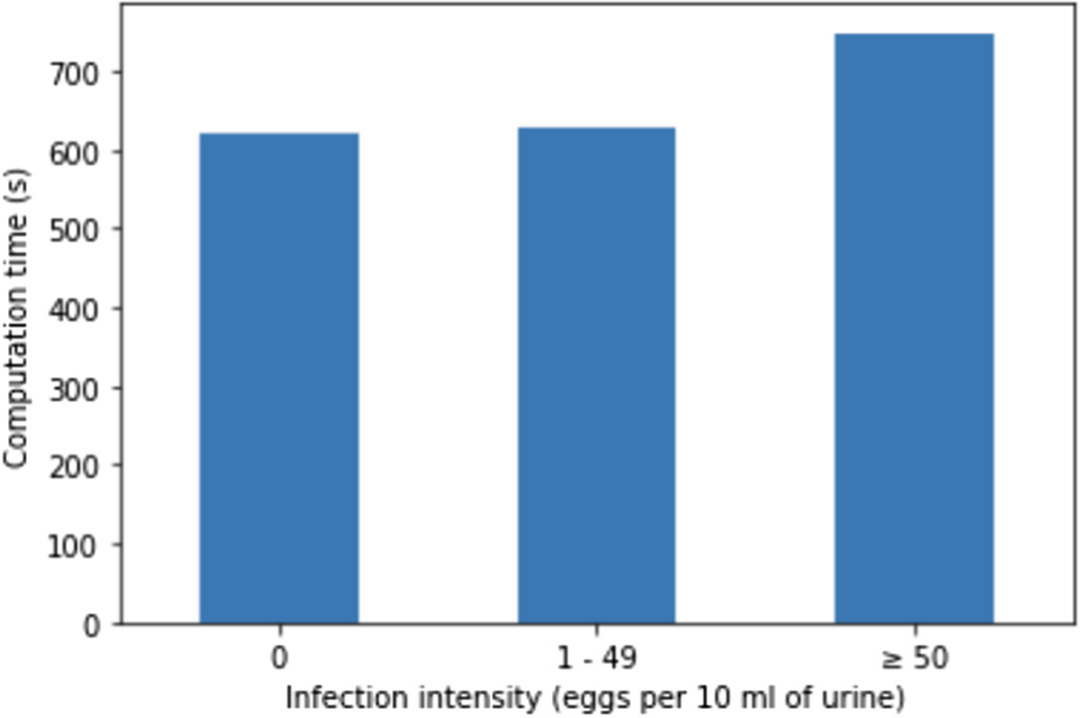
Average computational time in seconds from the application of framework on the diagnosis test image dataset.

To evaluate the computational performance of the developed framework, we measured the computational time of both stages of the proposed method as function of infection intensity. *S. haematobium* infection intensity has consistently been characterized by the number of schistosome eggs per 10 mL of urine with 1 to 49 eggs per 10 mL of urine defining a light infection and more than 50 eggs per 10 mL of urine indicating a heavy infection.^35^ We performed the running time experiments on a Raspberry PI 4B with Coral USB accelerator to study how the infection intensity affects the computational time. The algorithm was applied on images from the diagnosis test image dataset. Figure 8 shows the average computational time from the application of the first (DeeplabV3-MobileNetV3 semantic segmentation) and the second (refined segmentation and separation of overlapping eggs) stages of the developed framework to the diagnosis test image dataset as a function of the infection intensity. From this figure, it can be seen that there is little difference between the computational time of negative and light intensity samples (620 and 628 s, respectively). However, processing samples with heavy infection intensity is more time-consuming with an average computational time of 748 s.

## 6 Discussion

### 6.1 Impact on Schistosomiasis Control and Elimination

Schistosomiasis affects about 252 million people globally^2^ with ∼90% of infections and the vast majority of morbidity occurring in Sub-Saharan Africa. Chronic urogenital schistosomiasis infection can result in bladder fibrosis as well as female and male genital schistosomiasis, which is associated with greater risk of HIV transmission.^36^ Also the bulk of the more than 1.6 million disability-adjusted life years^37^ caused by schistosomiasis worldwide affect children, who have the highest prevalence and intensity of infections. Morbidity in children include anaemia, delays in physical and cognitive development, and reduced tolerance to exercise.^38^ The main strategy for control of schistosomiasis focuses on mass drug administration (MDA) of praziquantel in priority to primary school-aged children because it is more cost-effective to treat all school-aged children in a community above a certain prevalence threshold than to test and treat each individual.^4^ On a population level, higher intensities of infection are associated with higher levels of morbidity, but these relationships are poorly defined, as most control programs monitor only prevalence of infection and not intensity.^39^ Microscopic examination of urine samples is often a cheap and simple procedure recommended by WHO for the diagnosis of urogenital schistosomiasis. However, it has some critical shortcomings, which include access to microscopes and trained personnel as well as poor sensitivity and reproducibility, and an error-prone manual read-out.^40^ This led to the recent formation of the WHO Diagnostic Technical Advisory Group with the mandate to identify and prioritize diagnostic needs and to subsequently develop TPPs for future diagnostics.^4^^,^^41^^,^^42^ The TPP requires new diagnostic tools to have high specificity so as reliably measure when prevalence is above or below a cut-off of 10% in school-aged children. This informs decision on the frequency of the MDA. A diagnostic tool with high specificity is also needed to track changes of prevalence, ensuring that MDA is reducing overall prevalence, and to determine if transmission has been interrupted. In this work, we developed a two-stage diagnostic framework, which is a suitable candidate for estimating infection intensity and diagnostic prevalence in urogenital schistosomiasis monitoring and control.

### 6.2 Limitations

• *Image auto-focusing*. Some of the images in the dataset captured by the Schistoscope were blurry due to sub-optimal autofocusing. Although this had no effect on the diagnostic performance, it did have an effect on the automated egg counts of a few samples in the diagnosis test dataset. This problem has been solved by a more accurate auto-focusing algorithm in subsequent version of the Schistoscope.
• *Annotation problem*. Annotating the exact boundaries of the eggs was difficult due to their small sizes. This may contribute to the difficulty of the model to segment the exact egg boundaries, especially in overlapping eggs.
• *Diagnostic prevalence*. The determining diagnosis of a sample with eggs that are either broken or are at the edges of the images is mostly not considered by the developed framework as they do not meet the boundary requirements. This increases the chances of a false negative diagnosis especially in samples with very low egg counts.
• *Computational time*. On a Raspberry Pi with Coral USB accelerator, the developed framework processes 117 images of the 13 mm urine filter membrane in ∼11  min. Therefore, an estimated processing time of 35 min required to process a 25 mm filter membrane with 372 captured FoV images. However, the processing time can halved by the use of 2 Coral USB accelerators for computation through multi-threading.

## 7 Conclusion

We created a robust dataset of manually annotated *S. haematobium* eggs in microscopy images of urine samples collected from an endemic population, captured by the Schistoscope V5.0 device. We then developed a two-stage diagnosis framework for urogenital schistosomiasis using the SH dataset. The framework consists of two main stages, the first step involves the semantic segmentation of the eggs using the DeepLabV3 deep learning architecture with a MobileNetV3 backbone. The model effectively segmented the transparent eggs having low contrast with the background, and it also differentiated between eggs and other urine artifacts, such as crystals that have egg-like structures. In the next stage, a refined segmentation algorithm was applied to detect and count the eggs present. The refined segmentation algorithm separates overlapping eggs by fitting the region image with an optimal number of ellipses determined by optimising the AIC criterion. For improved diagnostic performance, we determine a sample as positive only if there is a detected egg present in the sample images that meet a defined boundary requirement, which is a function of the overlap and area of the fitted ellipse. We implemented the developed framework on an Edge AI system consisting of a Raspberry Pi 4B with Coral USB accelerator and applied it to a diagnosis test dataset of 65 samples using results obtained by an expert microscopist as reference. We obtained 93.75%, 93.94%, and 93.75% sensitivity, specificity, and precision, respectively. The automated egg count was also highly correlated with the manual count of the microscopist. The framework also provides causality for its estimated egg counts, which is relevant for diagnosis. From our results, it is evident that our automated framework for urogenital diagnosis combined with the Schistoscope device is a promising diagnostic tool for schistosomiasis. In a future study, the proposed multilayer framework, combined with the Schistoscope, will be validated for the diagnosis of urogenital schistosomiasis by comparing its performance with conventional microscopy as well as more accurate diagnostic methods, such as schistosome circulating antigen detection and DNA-based methods, such as polymerase chain reaction assays.^43^

## Data Availability

Schistosoma haematobium image dataset is available from the Zenodo Repository: https://doi.org/10.5281/zenodo.6467268.
